# Shared Decision Making and Patient-Centered Care in Israel, Jordan, and the United States: Exploratory and Comparative Survey Study of Physician Perceptions

**DOI:** 10.2196/18223

**Published:** 2020-08-03

**Authors:** Yaara Zisman-Ilani, Rana Obeidat, Lauren Fang, Sarah Hsieh, Zackary Berger

**Affiliations:** 1 Department of Social and Behavioral Sciences College of Public Health Temple University Philadelphia, PA United States; 2 Faculty of Nursing Zarqa University Zarqa Jordan; 3 Johns Hopkins School of Medicine Baltimore, MD United States

**Keywords:** shared decision making, patient-centered care, Middle East, physicians, perceptions

## Abstract

**Background:**

Shared decision making (SDM) is a health communication model that evolved in Europe and North America and largely reflects the values and medical practices dominant in these areas.

**Objective:**

This study aims to understand the beliefs, perceptions, and practices related to SDM and patient-centered care (PCC) of physicians in Israel, Jordan, and the United States.

**Methods:**

A hypothesis-generating comparative survey study was administered to physicians from Israel, Jordan, and the United States.

**Results:**

A total of 36 surveys were collected via snowball sampling (Jordan: n=15; United States: n=12; Israel: n=9). SDM was perceived as a way to inform patients and allow them to participate in their care. Barriers to implementing SDM varied based on place of origin; physicians in the United States mentioned limited time, physicians in Jordan reported that a lack of patient education limits SDM practices, and physicians in Israel reported lack of communication training. Most US physicians defined PCC as a practice for prioritizing patient preferences, whereas both Jordanian and Israeli physicians defined PCC as a holistic approach to care and to prioritizing patient needs. Barriers to implementing PCC, as seen by US physicians, were mostly centered on limited appointment time and insurance coverage. In Jordan and Israel, staff shortage and a lack of resources in the system were seen as major barriers to PCC implementation.

**Conclusions:**

The study adds to the limited, yet important, literature on SDM and PCC in areas of the world outside the United States, Canada, Australia, and Western Europe. The study suggests that perceptions of PCC might widely differ among these regions, whereas concepts of SDM might be shared. Future work should clarify these differences.

## Introduction

Shared decision making (SDM) is a central health communication model for supporting patient engagement in health care [1-3] and a recommended approach to increasing patient engagement and patient-centered care (PCC) in clinical decision making [4-6]. SDM evolved in Europe and North America [7] and largely reflects the values and medical practices dominant in these areas [8,9]. Although SDM has become more widely discussed in recent years in non-Western countries (eg, China, Peru, Malaysia, Taiwan, Iran) [10], it has yet to be implemented on a wider scale, and less is known about how or whether attitudes, beliefs, and practices regarding PCC exist or differ in various other regions of the world.

The overall aim of the present exploratory study was to explore the factors that enable or impede SDM implementation in different geographical and political contexts. Specifically, we sought to conduct a hypothesis-generating study and to collect preliminary data to better understand SDM- and PCC-related beliefs, attitudes, and practices of physicians in four regions in the Middle East characterized by different health care systems, cultures, and political environments: Israel, Jordan, and the West Bank. As a point of reference, we conducted a similar survey among US physicians to serve as a benchmark for SDM- and PCC-related beliefs, attitudes, and practices. In addition, such a comparison may provide insights about the importance of a health care system that facilitates the practice of SDM and PCC. The study focused on the following specific questions: (1) What are physicians’ common understandings and perceptions of the concepts of SDM and PCC? and (2) Do physicians find SDM and PCC to be feasible in their practice and in health care?

## Methods

### Settings: Context of Participating Countries

The survey was intended to be administered to physicians from different geographical and political contexts in the Middle East characterized by different health care systems: Israel, Jordan, and the West Bank. Israel is a democratic state with an efficient health care system that has been ranked among the top 10 health care systems for several years [11,12]. Israel’s national health insurance system provides universal health coverage throughout the country, with a significant spread of hospital and clinics [13-15]. Residents can supplement the universal coverage with additional forms of private health insurance. Israel’s health policy legislation is supportive of SDM principles, including the right to be informed of treatment options and risks [16]. Jordan is a constitutional monarchy state with a health care system characterized by diverse types of payers (public, private, and donors) [17]. The public health care sector is the largest in Jordan; however, only about 70% of residents have some form of public health insurance. Jordan has a ratio of 2.3 physicians to 1000 residents, and hospitals are mostly centralized in the larger urban areas. The massive influx of Syrian refugees due to the start of the Arab Spring in 2011 has further increased burdens on the Jordanian health care system, especially on public health facilities. The West Bank is an independent Palestinian territory governed by the Palestinian National Authority. The West Bank has low-functioning, inefficient health care systems that rely heavily on medical services to and referrals of patients to Israel (14% in 2011) or Jordan (13% in 2011) [17,18].

A parallel survey was planned among US physicians to serve as a benchmark. The United States is a representative democracy with a hybrid health care system but without universal health care coverage. In 2016, 48% of US health care spending came from private funds, with 28% coming from households and 20% coming from private businesses. The federal government accounted for 28% of spending, while state and local governments accounted for 17% of spending [19]. SDM in the United States is increasingly recognized as part of value-based care, and several federal initiatives have linked SDM to reimbursement [20].

### Survey Development and Structure

Because the purpose of the present study was to explore aspects of SDM and PCC and to provide data for testing hypotheses, we chose to develop a survey as a research strategy [20]. As recommended by Enhancing the Quality and Transparency of Health Research (EQUATOR), we used “Good Practice in the Conduct and Reporting of Survey Research” as a reporting guideline [20]. We developed a short standardized survey form with 24 questions divided into 3 parts (see Multimedia Appendix 1): (1) demographic data, (2) qualitative evaluation, and (3) quantitative evaluation.

First, demographic data (eg, age and years in practice) was assessed with 9 questions.

Second, a qualitative evaluation was included. One part of this evaluation assessed the beliefs and attitudes of physicians around PCC and SDM in their health care setting (eg, “What do you see as barriers to implementing shared decision making in your practice?”). This comprised 5 open-ended questions and 1 multiple choice question. The second part of the qualitative evaluation assessed the understanding of physicians of their immediate environment of practice in the context of their larger health care system (eg, “What are the most important day-to-day problems in the practice of medicine or health care in your country or region?”). This included 3 open-ended questions.

Third, the quantitative evaluation of level of SDM practice was based on the Shared Decision Making Questionnaire, physician version (SDM-Q-DOC) scale [21]. This included 9 questions on a 6-point Likert scale ranging from “completely disagree” (0) to “completely agree” (5). The SDM-Q-DOC was developed to measure patients’ and clinicians’ agreement with steps and actions defined by a medically driven SDM model at the end of a medical consultation. It has been used in numerous studies to measure physicians’ perspectives of SDM and was recommended for use in health policy survey responses targeting the implementation of SDM [22-24]

The qualitative part of the survey was developed through an iterative process based on SDM and PCC literature related to the delivery and perception of care and on the lead investigators’ (YZI and ZB) knowledge [25-30]. The development process included discussions among the coinvestigators and piloting among colleagues. We conducted forward and backward translations based on accepted guidelines [31] to each new question in sections 1 and 2. We used the English version of the SDM-Q-DOC questionnaire [21] and the Arabic [32] and Hebrew [33] translations of the 9-item Shared Decision Making Questionnaire (SDM-Q-9), a parallel patient version [34], with the needed minor adaptions.

### Procedure

A web-based survey developed for the study was emailed to physicians in Israel, the West Bank, and the United States using the Qualtrics platform (Qualtrics International Inc). In Jordan it was advised by one of the coauthors (RO) to administer the survey via face-to-face interviews, based on her previous experience conducting similar types of research in Jordan. A snowball sampling methodology was used to recruit physicians. Accordingly, participants were asked to identify and email the questionnaire to other colleagues. Surveys were administered in Hebrew, English, and Arabic, and all responses were anonymized. Data collection began in February 2017 and ended in June 2017. It was designed to stop after a sample of 15 in each country or after the maximum sample size closest to this threshold. As this was an exploratory study, this sample size target was based not on statistical considerations but on real-world experience with the number of respondents likely to provide a hypothesis-generating set of responses. At the end of the survey, participants were reimbursed via gift cards in the amount of US $10 or an equal value in the local currency.

### Data Analysis

To summarize the qualitative results from the open-ended survey questions, we used an integrated approach that enabled both inductive (ie, data-driven) coding of participants’ responses and deductive (ie, theory-driven) framework organization of codes [35]. Specifically, 2 coauthors (SH and ZB) and another research assistant read open-ended responses from participants and developed a draft of coding categories based on the responses’ contents. These categories were reviewed by the lead author (YZI) and revised accordingly. Responses to open-ended questions were coded independently by all coauthors; differences and disagreements between the coders were resolved through discussions until consensus was achieved. The final coding of open-ended responses was double-checked for accuracy by the first and last authors (YZI and ZB) after finalization of the coding guide. Then, guided by SDM and PCC theories, all coauthors discussed the interrelationships between codes to finalize the grouping of the codes into themes and subthemes.

Chi-square and one-way ANOVA tests were conducted to describe demographic characteristics of the survey respondents. As recommended by the developers [21], multiplication of the raw score by 20/9 provided a transformed total score range from 0 to 100, where 0 indicates the lowest possible level and 100 indicates the highest possible level of SDM. A nonparametric Kruskal-Wallis test was conducted to compare the mean total score of SDM-Q-9 between the 3 countries. Results were considered significant below a *P* value of .05.

### Ethics, Consent, and Permissions

Because the responses were anonymized and not identifiable and participation in the study was associated with minimal risk, the Institutional Review Board of the Johns Hopkins School of Medicine deemed this study exempt from requirements for approval (IRB00111847). All participants provided consent to participate through their responses to the survey.

## Results

### Participants

Eligible survey respondents were practicing physicians in the United States, Israel, Jordan, and the West Bank. A total of 36 survey responses were received from Israel (n=9), Jordan (n=15), and the United States (n=12). Throughout the study period, we received no responses from West Bank physicians to our emails or to our in-person attempts to contact them; therefore, we were unable to collect any data from that population. Most survey respondents were men (24/36, 67%), the mean age of survey respondents was 43.6 years (SD 11.2), and the mean years of clinical experience was 15.8 (SD 10.5). Comparison of demographic characteristics and clinical experience of clinicians in each country indicate similarity between US and Israeli respondents for gender distribution, age, and clinical experience (Table 1). The Jordanian respondents were significantly younger and less experienced, and almost all were men.

**Table 1:** Demographic and clinical experience characteristics of survey respondents.

Characteristics			Total sample (N=36)		United States (n=12)		Israel (n=9)		Jordan (n=15)		*P* value
**Gender**											.01^a^
	Men, n (%)	5 (42)		24 (67)		5 (56)		14 (93)		—^b^	
	Women, n (%)	7 (58)		12 (33)		4 (44)		1 (7)		—	
Age (years), mean (SD)			43.6 (11.2)		47.0 (8.6)		48.7 (13.4)		37.4 (9.2)		.02^c^
Years of clinical experience, mean (SD)			15.8 (10.5)		19.0 (9.8)		20.0 (14.2)		10.8 (6.0)		.05^d^

^a^Pearson *χ^2^*_2_=8.7.

^b^Not applicable.

^c^Analysis of variance *F* test (*F*_2,32_=4.40).

^d^Analysis of variance *F* test (*F*_2,33_=3.37).

### Open-Ended Responses: Perception of SDM and PCC

We included in the analysis 34 survey responses with greater than 50% total completion (Jordan: n=15; United States: n=12; Israel: n=7). There were 12 responses to the open-ended questions from the US physicians, 7 from the Israeli physicians, and 14 from the Jordanian physicians.

Most respondents defined SDM as a process aimed at informing patients (United States: 8/12, 67%; Israel: 4/7, 57%; Jordan: 12/15, 80%). Whereas most US respondents also defined SDM as the participation of patients in their care (8/12, 67%), only a third of the Jordanian respondents defined it as patient participation (5/15, 33%), and 4 of the 7 (57%) Israeli respondents defined SDM also as collaboration between patient and physician.

[SDM is when] a patient makes decisions about medical tests and treatment that incorporate information about benefits and harms from the physician as well as the patient’s own understanding of his or her values and priorities.

US respondent

[SDM is] an open conversation with the patient, in which I [the doctor] suggest/advise a variety of treatment options that fit the patient’s medical condition, and together with the patient, choose the appropriate treatment method.

Israeli respondent

[SDM is] giving the patient information about his treatment options and his illness and giving him a chance to have a say in his treatment options.

Jordanian respondent

Most US and Israeli respondents indicated familiarity with the concept of PCC (Israel: 5/7, 71%; United States: 10/12, 83%), whereas only 6 of the 15 (40%) Jordanian respondents indicated their or their patients’ familiarity with the concept. Prioritizing or meeting patient needs was commonly described as a feature of PCC by most respondents regardless of country of origin. In addition, US respondents commonly described PCC as accounting for patients’ preferences, most Israeli respondents described PCC also as individualized care, and most Jordanian respondents also described PCC as a provision of holistic care.

[PCC refers to] care that balances the needs and desires of the person receiving care.

US respondent

[PCC aims] to provide the patient with all of the patient’s needs and not just to solve a problem in the field, while maintaining proper communication and respect for the patient’s values.

Israeli respondent

[PCC refers] to doing whatever is needed for the patient or referring him/her to someone who can.

Jordanian respondent

Respondents indicated several barriers affecting the provision of SDM and PCC systematically; however, the common barrier was related to the system itself. All US respondents (12/12, 100%) mentioned lack of time as a major barrier to SDM implementation, whereas only 5 of 12 (42%) mentioned it as a barrier to PCC implementation. The role of insurance companies and fragmentation of care were mentioned as additional possible barriers to PCC implementation. In Jordan, most respondents (9/15, 60%) mentioned patient-related barriers, low health literacy, and a lack of knowledge as barriers to SDM implementation, whereas system-related barriers, such as staff shortages and high patient loads, were identified as barriers to PCC.

Time, and often hard to do for many decisions: few are really straightforward. Would be nice to have tools readily available to do this & ways to facilitate it.

US respondent, regarding SDM barriers

Lack of knowledge among patients and patient unwillingness to be fully informed about his/her condition.

Jordanian respondent, regarding SDM barriers

The healthcare delivery system is still organized traditionally regarding appointment scheduling and how patients interact with doctors; short visits limit person-centered care.

US respondent, regarding PCC barriers

Bureaucracy of the Jordanian health care system and lack of medical specialties in the peripheral areas of the country.

Jordanian respondent, regarding PCC barriers

With respect to problems related to the patient-physician relationship, the responses of Israeli physicians emphasized a lack of time and training (eg, lack of time, lack of support for physicians during their work). Jordanian respondents emphasized disorganization of the health care system, and US physicians highlighted problems of cost, social determinants of health, and the role of insurance companies (eg, the payment system and its incentive structure, lack of universal health care, costs of pharmaceuticals).

### SDM-Q-DOC Responses: Comparison of SDM Practice and PCC Behaviors

**Figure 1:**
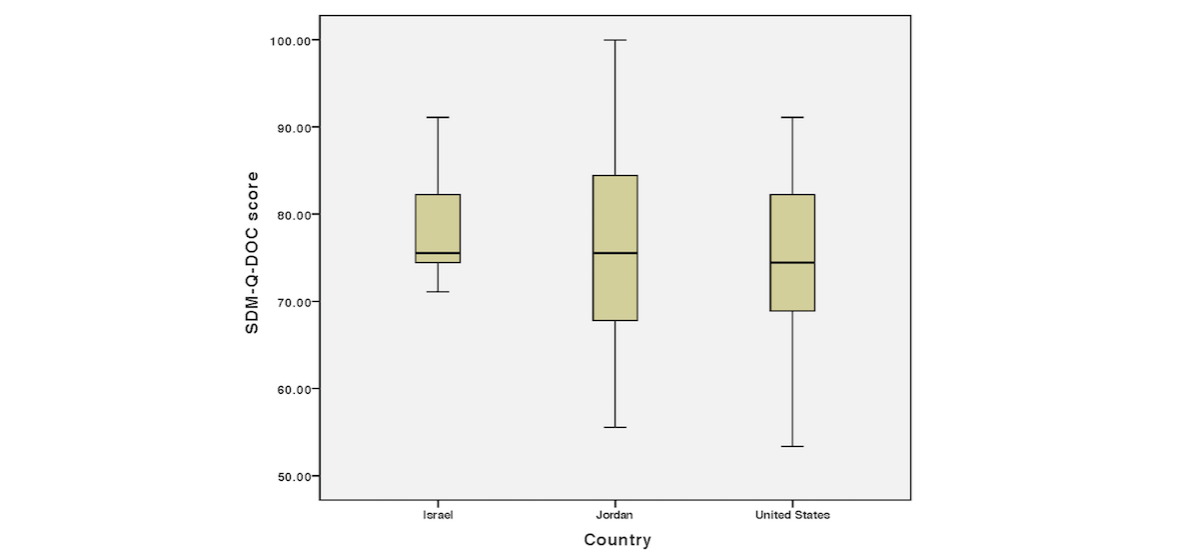
Box plot diagrams of SDM-Q-DOC score per country. SDM-Q-DOC: Shared Decision Making Questionnaire, physician version.

We included in the analysis 32 survey responses with greater than 50% total completion (Jordan: n=15; United States: n=10; Israel: n=7). Overall, physicians in our sample reported practicing SDM at a moderately high level (mean 76.6, SD 11.5; median 75.6), with a range of 53 to 100. This result is similar to findings in other studies [22]. The Kruskal-Wallis test results showed no significant difference in SDM-Q-DOC scores between the 3 countries (H_2_=0.631, *P*=.73; United States: mean 74.9, SD 11.4; Israel: mean 78.7, SD 7.9; Jordan: mean 76.7, SD 13.4). Box plot diagrams of SDM-Q-DOC means and standard deviations imply that SDM practice and PCC behaviors vary more among the US and Jordanian respondents in our sample than among their Israeli counterparts, as seen in Figure 1.

In addition, we compared the individual item scores between respondents from the 3 countries. Only for the first item (“I make clear to my patient that a decision needs to be made”) was a significant difference noticed, with higher scores for Jordanian physicians (Table 2). A nonsignificant but notable difference was noticed in the second item (“I want to know exactly from my patient how he/she wants to be involved in making the decision”), with a higher mean score for Jordanian physicians.

**Table 2:** Comparison of means and standard deviations of Shared Decision Making Questionnaire, physician version responses among respondents from the United States, Israel, and Jordan (n=32).

SDM-Q-DOC^a^ item question	H statistic	*P* value	United States, mean (SD)^b^	Israel, mean (SD)^b^	Jordan, mean (SD)^b^
1. I make clear to my patient that a decision needs to be made.	9.55	.008	3.60 (0.84)	3.57 (0.98)	4.47 (0.52)
2. I want to know exactly from my patient how he/she wants to be involved in making the decision.	4.66	.10	3.00 (0.67)	3.29 (0.76)	3.73 (0.80)
3. I tell my patient that there are different options for treating his/her medical condition.	1.83	.40	4.50 (0.71)	4.43 (0.98)	4.21 (0.58)
4. I precisely explain the advantages and disadvantages of the treatment options to my patient.	2.59	.27	3.80 (0.92)	4.43 (0.53)	4.13 (0.64)
5. I help my patient understand all the information.	2.06	.36	3.80 (0.92)	4.29 (0.76)	4.27 (0.96)
6. I ask my patient which treatment option he/she prefers.	1.00	.61	4.10 (0.74)	4.00 (1.00)	3.73 (0.96)
7. My patient and I thoroughly weigh the different treatment options.	2.53	.28	3.40 (0.52)	4.00 (0.82)	3.47 (0.92)
8. My patient and I select a treatment option together.	0.23	.89	3.50 (0.53)	3.43 (0.79)	3.13 (1.46)
9. My patient and I reach an agreement on how to proceed.	1.47	.48	8.84 (0.76)	4.00 (5.80)	3.67 (0.90)

^a^SDM-Q-DOC: Shared Decision Making Questionnaire, physician version.

^b^Means represent level of agreement with each item on a 6-point Likert scale, where 0=completely disagree and 5=completely agree.

## Discussion

### Principal Results and Comparison With Prior Work

The present study describes findings of a small, exploratory hypothesis-generating survey [20] of Israeli, Jordanian, and US physicians’ perceptions of SDM and PCC. Open-ended qualitative results suggest that respondents, regardless of country of origin, identify SDM as a process focused on providing information or as informed decision making, but PCC as a physician’s effort to meet patients’ individualized needs. These findings are aligned with the perception that SDM is “the pinnacle of PCC” [4] but also emphasize that SDM remains commonly perceived by physicians as a mean for delivering information rather than a collaborative discussion [36,37]. Barriers to implementing SDM and PCC were also identified and attributed to system- and patient-related factors [25]. The quantitative results of the total mean score of the SDM-Q-DOC show medium to high levels of SDM-related behaviors among all respondents. Item-focused analysis showed that Jordanian respondents scored significantly higher on item 1 (“I make clear to my patient that a decision needs to be made”), with a nonsignificant but notable difference for item 2 (“I want to know exactly from my patient how he/she wants to be involved in making the decision”). These are interesting results for the psychometric quality of the SDM-Q-DOC. Although there is less literature on the psychometric qualities of the SDM-Q-DOC, ample literature exists on the psychometric characteristics of the SDM-Q-9, showing mixed results for item 1 and suggesting eliminating the item to improve the factorial structure [22]. In our small sample, item 1 served as a discriminate item.

### Strengths and Limitations

The present study has several strengths. To the best of our knowledge, this is the first transnational comparison of the perceptions and practices of physicians in the United States, Israel, and Jordan. This survey study provided a conceptual overview of physicians’ understanding of SDM and PCC as well as an evaluation of SDM- and PCC-related behaviors. Although SDM is a communication model and practice and PCC is considered the conceptual framework [6], the participating physicians’ interpretation and understanding of the two were different. SDM was generally perceived as a means for delivering information to patients, whereas PCC was commonly perceived as a method for meeting a patient’s individual needs. While US and Jordanian physicians in our sample interpreted SDM also as a patient-level process (ie, patient participation), Israeli physicians in our sample interpreted SDM as a dialogue- and dyadic-based process occurring between patient and physician. These insights can inform future research and education initiatives pertaining to SDM and PCC among physicians. Finally, a methodological strength is our ability to deliver surveys in Arabic, Hebrew, and English due to the multilingual expertise of our team.

There are also some limitations to note. Our sample is small and not random; thus, we are unable to make meaningful statistical inferences or to generalize our findings, decreasing the study’s validity. However, because the purpose of this exploratory study was to generate hypotheses, our snapshot of the SDM landscape and PCC perceptions among physicians in Israel and Jordan is new and will inform future scaled-up surveys. We surveyed physicians, not other members of the health care team or patients. Clearly, a future comprehensive comparison of these health systems and practitioners’ approaches to care requires surveying all stakeholders involved. The SDM-Q-DOC questionnaire was administered to explore physicians’ perceptions of what is important in an SDM encounter, but it was not used to evaluate an actual encounter. Although the SDM-Q-DOC was developed to rate physicians’ experiences of SDM in patient-physician encounters, recent literature indicates the feasibility of the Shared Decision Making Questionnaire family for use in surveys [23,28,38]. The final limitations are that we were unable to recruit and collect data from physicians in the West Bank and that the survey in Jordan was conducted using face-to-face interviews rather than web-based surveys. Using email to initiate communication with potential Jordanian and West Bank respondents was challenging, as was administering a web-based survey. In Jordan, we learned that recruitment by phone call or an offline survey methodology that does not require an internet connection could be better for collecting responses. Therefore, we employed face-to-face interviews successfully, but this might have caused a social desirability bias; that is, physicians might have overestimated their support for and use of the SDM approach. However, because the web-based platform was the tool rather than the purpose, we believe it was not critical and that the benefits of collecting data face to face instead of via a web-based survey were more important for this project.

### Conclusions

The results of the present study add to the limited, yet important, literature on SDM and PCC in areas of the world outside the United States, Canada, Australia, and Western Europe [9,12,39-43]. They also add to the psychometric evaluation of SDM and PCC measures [44] and identify barriers to implementation. We hope this survey will motivate researchers and clinicians in Israel, Jordan, and other countries that are less represented in the SDM and PCC research and practice to encourage related discussions and practice and to facilitate implementation, measurements, and interventions.

## Appendix

Multimedia Appendix 1 Physician Perceptions of Shared Decision Making and Person-Centered Care Survey.

## Abbreviations

EQUATOR: Enhancing the Quality and Transparency of Health Research
PCC: patient-centered care
SDM: shared decision making
SDM-Q-9: 9-item Shared Decision Making Questionnaire
SDM-Q-DOC: Shared Decision Making Questionnaire, physician version
