# Supplementary material for: Shared Decision Making and Patient-Centered Care in Israel, Jordan, and the United States: Exploratory and Comparative Survey Study of Physician Perceptions
Source: JMIR Form Res. 2020 Aug 3;4(8):e18223. doi: 10.2196/18223 (PMC7432149; doi:10.2196/18223)
Supplement: Multimedia Appendix 1 [file formative_v4i8e18223_app1.docx]

English-Physician Perceptions of Shared Decision Making and Person-Centered Care Survey

Start of Block: Survey Questions

Q1 Hello!
 
Thank you for agreeing to participate in our short, international survey about shared decision making practices in healthcare delivery. Your responses are anonymous and will help us better understand and facilitate policy and practice issues. The survey is expected to take 15 minutes to complete. Upon completion of the survey you will be offered a $5 Amazon gift card. 
 

Your completion of this survey will serve as your consent to be in this research study.
Please press the next button if you would like to continue.    

Q2 Gender

- Male (1)
- Female (2)

Q3 Age

- Please enter your age in years (1) ________________________________________________

Q4 Country

- Israel (1)
- Jordan (2)
- United States (3)
- West Bank (4)
- Gaza Strip (5)

Display This Question:

If Country = United States

Q5 Race

- Please enter your response here (1) ________________________________________________

Display This Question:

If Country = Israel

Or Country = Jordan

Or Country = West Bank

Q6 Religion

- Please enter your response here (1) ________________________________________________

Q7 How many years have you practiced medicine?

- Please enter your response here (1) ________________________________________________

Q8 What percent of your work week is spent seeing patients?

- 0 % (1)
- 1-25 % (2)
- 26-50 % (3)
- 51-75 % (4)
- 76-100 % (5)

Q9 Where did you train?

- Jordan (1)
- West Bank (2)
- Israel (3)
- Elsewhere in Middle East (4)
- Europe (5)
- Africa (6)
- United States (7)
- Other (please specify) (8) ________________________________________________

Q10 In what language do/did you practice?

- Hebrew (1)
- Arabic (2)
- English (4)
- Other (please specify) (3) ________________________________________________

Q11 What are the most important systematic problems relating to health in your country or region (e.g., types of care which need improvement or which are lacking; health care costs; quality of care; disadvantaged populations)?

________________________________________________________________

________________________________________________________________

________________________________________________________________

________________________________________________________________

________________________________________________________________

Q12 What are the most important day-to-day problems in your country or region regarding staying healthy or interacting with health care providers?

________________________________________________________________

________________________________________________________________

________________________________________________________________

________________________________________________________________

________________________________________________________________

Q13 What are the most important day-to-day problems in the practice of medicine or health care in your country or region?

________________________________________________________________

________________________________________________________________

________________________________________________________________

________________________________________________________________

________________________________________________________________

Q14 Can you define person (or patient) centered care in your own words?

________________________________________________________________

________________________________________________________________

________________________________________________________________

________________________________________________________________

________________________________________________________________

Q15 Are patients, providers, or caregivers in your region familiar with the concept of person-centered care?

- yes (1)
- no (2)
- not sure (3)

Q16 What do you see as barriers to implementing person-centered care in your practice?

________________________________________________________________

________________________________________________________________

________________________________________________________________

________________________________________________________________

________________________________________________________________

Q17 Can you define shared decision making in your own words?

________________________________________________________________

________________________________________________________________

________________________________________________________________

________________________________________________________________

________________________________________________________________

Q18 How familiar are patients, providers, or caregivers in your region with the concept of shared decision making?

________________________________________________________________

________________________________________________________________

________________________________________________________________

________________________________________________________________

________________________________________________________________

Q19 What do you see as barriers to implementing shared decision making in your practice?

________________________________________________________________

________________________________________________________________

________________________________________________________________

________________________________________________________________

________________________________________________________________

Q20 Please answer the questions about your practice and shared decision making. The questions refer to your usual practice with patients.

|  | Completely Disagree (1) | Strongly Disagree (2) | Somewhat Disagree (3) | Somewhat Agree (4) | Strongly Agree (5) | Completely Agree (6) |
| --- | --- | --- | --- | --- | --- | --- |
| I make clear to my patient that a decision needs to be made. (1) |  |  |  |  |  |  |
| I want to know exactly from my patient how he/she wants to be involved in making the decision. (2) |  |  |  |  |  |  |
| I tell my patient that there are different options for treating his/her medical condition. (3) |  |  |  |  |  |  |
| I precisely explain the advantages and disadvantages of the treatment options to my patient. (4) |  |  |  |  |  |  |
| I help my patient understand all the information. (5) |  |  |  |  |  |  |
| I ask my patient which treatment option he/she prefers. (6) |  |  |  |  |  |  |
| My patient and I thoroughly weigh the different treatment options. (7) |  |  |  |  |  |  |
| My patient and I select a treatment option together. (8) |  |  |  |  |  |  |
| My patient and I reach an agreement on how to proceed. (9) |  |  |  |  |  |  |

End of Block: Survey Questions
